# A genome-wide screen in *Saccharomyces cerevisiae* identifies Tannic Acid-sensitive mutants

**DOI:** 10.17912/micropub.biology.000358

**Published:** 2021-01-14

**Authors:** Emily M Pilc, Shriie Ganesh, Oliver Kerscher

**Affiliations:** 1 William & Mary, Biology Department, Williamsburg, VA 23187

**Figure 1. TA-sensitivity screen of the yeast deletion library and analysis of mutants:**
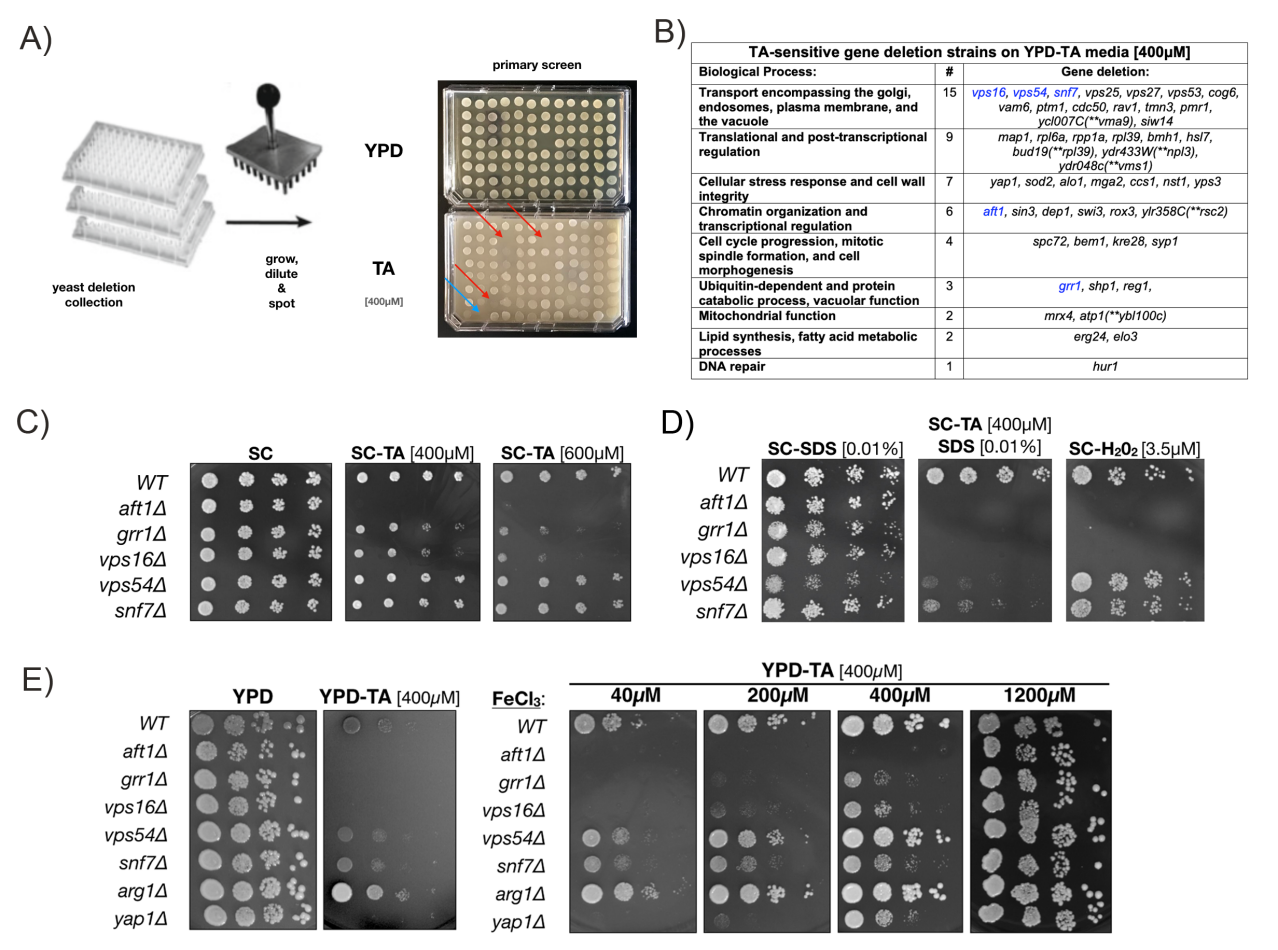
A)Schematic of the primary TA sensitivity screen: >5100 strains of the yeast deletion library were grown up in 96-well plates, diluted and spotted to YPD or YPD-TA plates to identify TA-sensitive deletion mutants (red arrows). A TA-sensitive *yap1∆* strain was included as a control on each plate (blue arrow). (B) Table with biological process categories of 49 yeast deletion strains identified in the TA-sensitivity screen on YPD-TA media. Individual gene deletions and number (#) in each category are indicated. The gene names in parentheses (indicated by **) identify overlapping genes used for biological process annotation. (C) TA growth assays of select gene deletion strains (shown in blue in table) and a WT control on SC or SC-TA media with 400 or 600µM TA. (D) TA growth assays of select gene deletions and a WT control on SC media with SDS, SDS and TA (400µM), or hydrogen peroxide (3.5µM). (E) TA growth assays of select TA-sensitive deletion strains (*aft1∆, grr1∆, vps16∆, vps54∆, snf7∆*) and controls (WT, *arg1∆, yap1∆*) on YPD or YPD-TA media supplemented with indicated concentrations of ferric chloride (FeCl_3_).

## Description

We conducted a genome-wide chemical genetics screen of the yeast MATa knockout collection to identify genes that are required for survival on sub-lethal levels of the polyphenol Tannic Acid (TA) **(see Fig 1A and reagents)**. TA, a plant-derived polymeric form of gallic acid connected to a central glucose core, is known to bind and precipitate proteins (Hagerman and Klucher 1986), chelate iron (ferric ions, Fe^3^), and form TA-Fe^3^ films on cells, including yeast (Lee *et al.* 2020). Addition of TA to cultured prostate cancer cells is reported to induce ER stress, increase markers of the unfolded protein response (UPR), and inhibit lipid metabolism (Nagesh *et al.* 2020). TA has also been reported to act as a potent but non-toxic SUMO E1 inhibitor in several cell lines (Suzawa *et al.* 2015). How TA induces these effects remains unknown and, therefore, the genome-wide TA-sensitivity screen may provide insights into underlying genetic pathways involved.

Our initial TA-sensitivity screen of the MATa yeast knockout collection (>5100 strains) netted 49 deletion strains that grew on rich YPD media but showed greatly reduced or no growth on the same media with 400µM TA (**Fig 1A**). In this screen a TA-sensitive *yap1∆* strain (carrying a deletion of the *YAP1* transcription factor required for oxidative stress tolerance) and a TA-resistant wildtype strain (BY4741) served as positive and negative controls, respectively. The majority of gene deletions identified in our TA-sensitivity screen fell into 6 categories: Vesicle-mediated transport (30.6%), translational and post-transcriptional regulation (18.4%), cellular stress response (14.3%), chromatin organization and transcriptional regulation (12.2%), cell cycle (8.2%), and catabolic processes (6.1%) **(Fig 1B).**

It has previously been reported that the sensitivity of *Saccharomyces cerevisiae* to TA is due to iron deprivation, but this finding has not been evaluated in the context of specific yeast deletion mutants (Wauters *et al.* 2011). Therefore, we repeated the growth assay with our 49 TA-sensitive deletion strains on Synthetic Complete (SC) media with and without 400µM TA. SC media contains an almost 50-fold higher ferric chloride concentration [~1200µM] than YPD media [~25µM]. Unexpectedly, most TA-sensitive deletion strains grew similar to the wildtype control on SC and SC-TA media, with 5 notable exceptionsdiscussed in detail below **(**highlighted in table **Fig.** **1B** and Fig. **1C)**. First, a *aft1∆* deletion strain failed to grow on SC media with both 400 and 600µM TA. Second, *grr1∆* and *vps16∆* deletions showed a concentration-dependent reduction in growth on 400 and 600µM TA. Third, *vps54∆* and *snf7∆* deletion strains grew unperturbed on SC with 400 and 600µM TA, but became TA-sensitive when the media was supplemented with 0.01% SDS (Sodium dodecyl sulfate), an ionic detergent. SDS addition alone did not affect growth of these deletion strains and hydrogen-peroxide (H_2_O_2_) plates were included as controls to assess oxidative stress tolerance **(Fig. 1D).**

Next we confirm that increased levels of ferric chloride (FeCl_3_) permitted growth of the TA-sensitive deletion mutants identified in our assay (**Fig. 1C**). As expected from our initial screen, *aft1∆*, *grr1∆*, *vps16∆, vps54∆* and *snf7∆* grew robustly on YPD media but showed little or no growth on YPD-TA media (**Fig. 1E – left**). However, when YPD-TA plates were supplemented with increasing levels of FeCl_3_ (40-1200µM), growth defects of these 5 TA-sensitive mutants improved in a concentration-dependent manner and no difference to wildtype cells was detected at the highest concentration (1200µM) (**Fig. 1E – right**). A wildtype strain (WT), the TA-sensitive *yap1∆* strain, and a TA-tolerant deletion of the arginosuccinate synthetase(*arg1∆*), were included as controls. These results indicate that iron depletion is the causative factor for the TA-sensitivity of these yeast deletions.

Iron is an essential element and its depletion affects numerous biological processes. For example, during severe iron deficiency both cellular transcription and translation are repressed (Romero *et al.* 2020). *AFT1* encodes a transcriptional activator that regulates iron uptake and storage genes, which readily explains the TA-sensitivity of the *aft1∆* deletion mutant in our screen as it fails to regulate iron homeostasis. Amongst the TA-sensitive mutants *aft1∆* is the most sensitive both on SC-TA and YPD-TA media. However, it is currently not clear why addition of FeCl_3_ rescues the growth defect of *aft1∆* on YPD-TA (**Fig. 1E**) but not SC-TA (**Fig. 1C**). Therefore, the conclusion that iron supplementation can generally rescue a TA-induced growth defect of *aft1∆* is preliminary. Both the *aft1∆,* as well as the *snf7∆* strain, were also previously identified as sensitive to iron depletion from yeast cultures supplemented with BPS (Bathophenanthrolinedisulfonic acid), another iron chelating agent (Jo *et al.* 2009). Snf7 and Vps16, as well as Vps54, are involved in transport encompassing the golgi, endosomes, and the vacuole, supporting normal function of the vacuole in storage and sequestration of iron and toxic metabolites (Bode *et al.* 1995; Jo *et al.* 2009; Zhou *et al.* 2009). This suggests that either iron sequestration or TA detoxification may be defective in these mutants. However, it is important to note that while TA associates with the surface of yeast cells, there is currently no evidence showing the intracellular uptake of this polyphenol or its metabolites (Lee *et al.* 2020). Grr1, in contrast, is an F-box substrate adaptor protein of the SCF ubiquitin ligase complex and involved in cell cycle progression. Notably, *grr1∆* mutants display an abnormal cell shape and exhibit increased sensitivity to osmotic stress (Flick and Johnston 1991). Our observation that low concentrations of SDS exacerbate the effect of TA in the *grr1∆* (and our other mutants) may indicate that TA treatment exacerbates membrane integrity defects. There are several reports indicating that TA exposure negatively affects lipid biogenesis and membrane composition (Wauters *et al.* 2011; Nagesh *et al.* 2020). Finally, it is important to note that the TA growth phenotype on SC-TA media is similar to those observed on media supplemented with hydrogen peroxide (**Fig. 1D**), suggesting that oxidative stress and the oxidative damage of proteins and lipids may also play a role in TA’s effect.

In summary, our data shows that TA-sensitivity for the indicated yeast cell mutants can be remedied by ferric chloride supplementation to the growth media (**Fig. 1E**). This suggests that the 49 yeast deletion mutants identified in our screen fail to cope with TA-induced iron-deficiency stress. Considering TA-mediated iron depletion, there are at least 21 iron-ion binding proteins expressed in yeast that may be affected by reduced iron levels (Cherry *et al.*, 2012). These iron-binding proteins include those required for ribosome biogenesis and function (e.g. *RLI1*), lipid biogenesis (e.g. *SUR2*, *SCS7*, *ERG5* etc.), NAD biosynthesis (e.g. *BNA1*), mitochondrial function (e.g. *NFU1*), and iron chaperone activity (e.g. *YFH1*). Thus, TA’s iron-chelating activity in the growth media is likely to exert pleiotropic effects on eukaryotic cell growth which must be taken into account when evaluating studies conducted with this polyphenol.

## Methods

Primary Screen of the Yeast Deletion Library: Individual frozen strains of the yeast deletion strain collection in 96-well format were recovered from cold-storage and grown for 2-3 days as patches on solid YPD media with G418. Cells from individual patches were then inoculated into the corresponding wells of a 96-well plate with 200µl of liquid YPD media and allowed to grow for 2 days at 30°C. Cells were then resuspended, diluted 1:200 in water, and 5µl of each deletion strain was spotted on YPD and YPD-TA (400µM) media. Media plates with cell patches were imaged and data recorded after growing for 5 days at 30°C (see Fig.1A). Secondary Growth Assays: TA-sensitive clones were recovered and tested on the indicated media and with the appropriate controls by spotting 5µl of serially diluted cells (a 10-fold dilution for each spot) from a log phase grown culture. Media plates with colonies were imaged and data recorded after growing for 2-5 days at 30°C (see Fig 1. 1C-E). Biological Process information was assigned using information from www.yeastgenome.org (Cherry *et al.*, 2012) and Panther GO slim (pantherdb.org).

## Reagents

Yeast were grown and assayed at 30°C in YPD or synthetic-complete (SC) growth media with the stated additions (Guthrie and Fink 2004). Yeast nitrogen base for SC media was purchased from Sigma-Aldrich (Y0626). G418 (USBiological G1000) was added to 200µg/ml to select for individual *KANMX4*-marked gene deletion strains in the MATa knockout collection (Giaever *et al.* 2002). Strain genotype for the MATa deletion collection is: BY4741 *MATa his3∆1 leu2∆0 met15∆0 ura3∆0*) (Brachmann *et al.* 1998). Yeast deletion strains are available at https://horizondiscovery.com*.* Tannic Acid (TA) (Sigma-Aldrich 403040) was dissolved in water to 100mM and diluted in pre-warmed YPD or SC media at the indicated concentrations before pouring. FeCl_3 _was dissolved in water to 100mM and added to media at the indicated concentrations and before adding TA. Where indicated, hydrogen peroxide was diluted into media from a commercial stock to 3.5µM. SDS was diluted into media from a 20% stock in water to 0.01%.
